# Proteomic analysis of differential proteins in pancreatic carcinomas: Effects of MBD1 knock-down by stable RNA interference

**DOI:** 10.1186/1471-2407-8-121

**Published:** 2008-04-29

**Authors:** Chen Liu, Yaohui Chen, Xianjun Yu, Chen Jin, Jin Xu, Jiang Long, Quanxing Ni, Deliang Fu, Hong Jin, Chen Bai

**Affiliations:** 1 Pancreatic Disease Institute, Department of General Surgery, Huashan Hospital, Fudan University, Shanghai 200040, PR China; 2 Department of Biochemistry, Fudan University, Shanghai 200433, PR China

## Abstract

**Background:**

Methyl-CpG binding domain protein 1 (MBD1), a suppressor of gene transcription, may be involved in inactivation of tumor suppressor genes during tumorigenesis. Over-expression of MBD1 has been reported in human pancreatic carcinomas.

**Methods:**

In this study, we established a MBD1-knock-down pancreatic cancer cell line (BxPC-3) using stable RNA interference, to compare the proteomic changes between control and MBD1-knock-down cells using two-dimensional gel electrophoresis and mass spectrometry.

**Results:**

We identified five proteins that were up-regulated and nine proteins that were down-regulated. Most of the identified proteins are involved in tumorigenesis, some are prognostic biomarkers for human malignant tumors.

**Conclusion:**

Our data suggest that these differential proteins may be associated with the function of MBD1, and provide some insight into the functional mechanism of MBD1 in the development of pancreatic cancer.

## Background

The incidence rate of pancreatic cancer has increased significantly in recent years. Recent studies examining the origin of pancreatic cancer have revealed that molecular alterations, including changes in tumor suppressor genes and oncogenes involved in multiple cellular signaling pathways, may have a significant role in the multistage carcinogenesis of pancreatic cancer [1].

DNA methylation at CpG islands is the major epigenetic modification of mammalian genomes and is required for gene regulation and genome stability [2]. Aberrant DNA methylation, especially the hypermethylation of tumor suppressor genes, has been reported to be associated with the inactivation of tumor suppressor genes and tumorigenesis [3]. Methyl-CpG binding domain protein 1 (MBD1) is a mammalian protein that binds methylated CpG islands symmetrically and couples DNA methylation to transcriptional repression [4]. This biological property suggests a role for MBD1 in the silencing of tumor suppressor genes that may contribute to tumorigenesis [4,5]. We have previously reported that MBD1 is over-expressed in human pancreatic carcinomas and that over-expression of MBD1 correlated significantly with lymph node metastasis [6]. However, the role of MBD1 in the development of pancreatic cancer is still unknown.

In this study, we silenced MBD1 expression in the pancreatic cancer cell line BxPC-3 using the RNA interference (RNAi) technique. We used two-dimensional gel electrophoresis (2-DE) to detect differential protein expression in the BxPC-3/MBD1-siRNA and control BxPC-3/vector cell lines. The differential expression patterns between the two cell lines were identified by matrix-assisted laser desorption/ionization time-of-flight mass spectrometry (MALDI-TOF-MS). Our data provide some insight into the functional mechanism of MBD1 in the development of pancreatic cancer.

## Methods

### Cell lines and culture

The human pancreatic cancer cell line, BxPC-3, was purchased from Shanghai Institutes for Biological Science (China). Cells were cultured in RPMI-1640 media (Gibco BRL, USA) supplemented with 10% fetal bovine serum (FBS) (Gibco BRL, USA) in a 37°C incubator with 5% CO_2_.

### Construction of the recombinant MBD1-siRNA plasmid

The design of two double stranded siRNA oligonucleotides targeting MBD1 was based on the published sequence of MBD1 (BC033242). *BamH *I and *Hind *III recognition sequences were added as indicated below. The MBD1 target 1 sequence was 5'- GCATCTGGCCCAGGAATTA -3'. The forward oligonucleotide sequence was: 5'...GATCCCGCATCTGGCCCAGGAATTAttcaagagaTAATTCCTGGGCCAGATGC TTTTTTGGAAA ...3' and the reverse sequence was: 5'...AGCTTTTCCAAAAAA GCATCTGGCCCAGGAATTA tctcttgaaTAATTCCTGGGCCAGATGC GG ...3'. The MBD1 target 2 sequence was 5'- CCAAGAGGATTGTGGCCAT -3'. The forward oligonucleotide sequence was: 5'...GATCCCCCAAGAGGATTGTGGCCATttcaagaga ATGGCCACAATCCTCTTGG TT TTTTGGAAA ...3', the reverse sequence was: 5'...AGCTTTTCCAAAAAACCAAGAGGATTGTGGCCATtctcttgaaATGGCCACAATCCTCTTGGGG ...3'. The oligonucleotides were annealed in a buffer (100 mmol/L potassium acetate, 30 mmol/L HEPES-KOH pH 7.4, and 2 mmol/L Mg-acetate) and incubated at 95°C for 4 minutes, slow cooling to room temperature for 1 hour. The restriction endonucleases *BamH *I and *Hind *III were used to linearize the PGCsi-U6/Neo/GFP vector (kindly provided by Professor Huang Weida, Department of Biochemistry, Fudan University). The annealed double stranded oligonucleotides were ligated into the *BamH *I and *Hind *III sites of the linear pGCsi-U6/Neo/GFP vector using T4 DNA ligase. The plasmid was then transformed and recombinant plasmid DNA was extracted for DNA sequencing.

### Stable transfection

The targeting and control vectors were transfected into BxPC-3 cells using Lipofectamine 2000 (Invitrogen, USA). Briefly, BxPC-3 (80–90% confluence), were subcultured into 6-well plates (1 × 10^6 ^cells/well) at 37°C in a humidified atmosphere of 5% CO_2 _for 24 hours. The diluted plasmid and liposome were incubated in serum and antibiotics-free DMEM for 5–10 minutes then added to the cell culture plates. The transfected cells were cultured for 5 hours then transferred to fresh media containing 10% FBS. G418 was used to select the positive clones. BxPC-3 cells stably transfected with the MBD1-siRNA plasmid were named "BxPC-3/MBD1-siRNA". Control BxPC-3 cells transfected with vector alone were named "BxPC-3/vector".

### Western blot analysis

The total cell lysate was separated on a 10% sodium dodecyl sulfate-polyacrylamide gel using electrophoresis (SDS-PAGE) and transferred onto a polyvinylidene difluoride(PVDF) membranes. The membrane was blocked for 1 hour at room temperature in 10% FBS, then incubated overnight at 4°C with different primary antibodies (anti-MBD1, USBiological, USA, 1:250; anti-vimentin, Santa Cruz, USA, 1:250; anti-stathmin, Cell Signaling Technology, USA, 1:250;anti-hnRNP K Santa Cruz, USA, 1:500; anti-GRP78, BD Pharmingen, USA, 1:300; anti-HSP70, BD Pharmingen, USA, 1:300; anti-tubulin beta2, Novocastra, UK,1:50). After washing three times, the membrane was incubated with secondary antibody for 1 hour at room temperature. The signal was detected by the ECL detection system (Chemicon, USA)

### Real-time quantitative RT-PCR

Total RNA was extracted from the BxPC-3/MBD1-siRNA and BxPC-3/vector cells using Trizol reagent (Gibco BRL, USA). First-strand cDNA was synthesized from 1 μg total RNA using RevertAid™ M-MuLV Reverse Transcriptase (Fermentas, USA) according to the manufacturer's instructions. Quantitative RT-PCR was performed on an ABI PRISM 7300 system using SYBR Green PCR Master Mix (Takara, Japan). Primer sequences and annealing temperature for MBD1, vimentin and GRP78 were shown in Table 1. β-actin was used as an endogenous control.

**Table 1:** Quantitative RT-PCR primer sequences, amplicon length and annealing temperature

Gene	Primer sequences	Amplicon length	Annealing temperature
MBD-1	5' TCTGGTTGCCAAGGTCCAAA 3' 5' ACATCCATCTTCCCTTCCCGA 3'	122bp	61°C
vimentin	5' TGGAAGAGAACTTTGCCGTTG 3' 5' AAGGTGACGAGCCATTTCCTC 3'	101bp	60°C
GRP78	5' CACCAATGACCAGAATCGCCT 3' 5' CAATGCGCTCCTTGAGCTTT 3'	101bp	60°C

Quantification results were expressed in terms of the cycle threshold (CT) value. The comparative CT method [7] was used to quantify relative MBD1, vimentin and GRP78 expression. Briefly, the CT values were averaged for each triplicate. Differences between the mean CT values of MBD1, vimentin, GRP78 and those of β-actin were calculated as delta CT = CT (target gene) -CT (β-actin). Final results, expressed as N-fold differences of gene expression between BxPC-3/MBD1-siRNA and control BxPC-3 cells, were determined as 2^-delta delta CT ^(delta delta CT = delta CT BxPC-3/MBD1-siRNA – delta CT control BxPC-3).

### 2-D gel electrophoresis

BxPC-3/MBD1-siRNA and BxPC-3/vector cells were harvested, and lysed in lysis buffer (7 M urea, 2 M thiourea, 65 mM DTT, 4% Chaps, 40 mM Tris, 2% Pharmalyte). After incubation at 37°C for 1 hour, the lysates were centrifuged at 15000 rpm for 30 minutes at 4°C. The concentration of the total protein in the supernatant was determined using the Bradford method. Protein samples were diluted to 350 μl with rehydration solution (7 M urea, 2 M thiourea, 15 mM DTT, 0.5% IPG (immobilized pH gradient) buffer, and trace bromophenol blue) and applied to IPG strips (pH 4–7, Amersham Biosciences, Sweden) for 12 hours. Isoelectric focusing (IEF) was performed on an Ettan IPGphor (Amersham Biosciences). Focused IPG strips were equilibrated for 15 minutes in a solution (6 M urea, 2% SDS, 30% glycerol, 50 mM Tris-HCl, pH 8.8 and 1% DTT), followed by 15 minutes in the same solution containing 2.5% iodoacetamide instead of 1% DTT. Equilibrated strips were placed on 10% acrylamide gels containing SDS. SDS-PAGE was performed on a PROTEAN II system (Bio-Rad, USA). After electrophoresis, silver staining was performed as described previously [8].

### Image scanning and analysis

The stained 2-DE gels were scanned using a GS-800 Imagescanner (Bio-Rad), and analyzed using ImageMaster 2D software (Amersham Biosciences). Three separate gels with the fewest artifacts were prepared for each cell line and selected for statistical analysis. The criterion for a differential expression of any particular protein between the two cell lines was set as at least a 3-fold change in spot intensity according to a previous study [9].

### Protein identification by MALDI-TOF-MS

The protein spots were excised using an Ettan spot picker (Amersham Biosciences) and digested with trypsin. Briefly, silver-stained protein spots were destained with 15 mM potassium ferricyanide and 50 mM sodium thiosulfate (1:1) and washed with Millipore-Q water. The gel pieces were dehydrated with acetonitrile, dried in a vacuum centrifuge, and incubated in 20 μl of digestion solution containing 20 mM ammonium bicarbonate and 20 ng/μl sequencing grade trypsin (Promega, USA). After the tryptic digestion at 37°C for at least 3 hours, the resultant peptides were extracted (50 μl of 50% acetonitrile), desalted with ZipTip C_18 _columns (Millipore Corp, USA), and eluted with 2.5 μl of 50% acetonitrile containing 0.5% TFA (Triflouroacetic acid) and 3 mg/ml α-cyano-4-hydroxycinnamic acid. Samples were spotted onto stainless steel MALDI sample target plates, and analyzed by 4700 Proteomics Analyzer MALDI-TOF/TOF mass spectrometer (Applied Biosystems, USA) in the positive ion reflector mode. Peptide matching and a protein search against the NCBI databases were performed using the Mascot search engine.

### Statistical analysis

Student's *t *test was used to compare the difference between mean values. *P *< 0.05 was considered to be statistically significant. All statistical calculations were performed using the SPSS11.5 statistical software package.

## Results

### Establishing stable MBD1-knock-down pancreatic carcinoma cell lines

**Figure 1:**
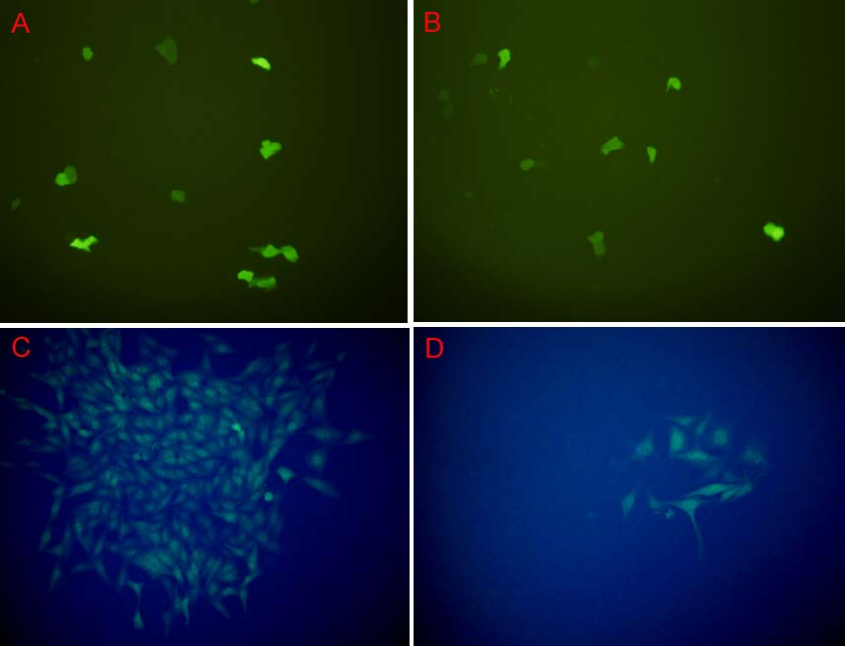
**Stable transfection of the MBD1-siRNA recombinant plasmid in BxPC-3 cells**. A, B: Transfected cells 24–72 hours after transfection. C, D: Positive cell clones 3–4 weeks after transfection.

**Figure 2:**
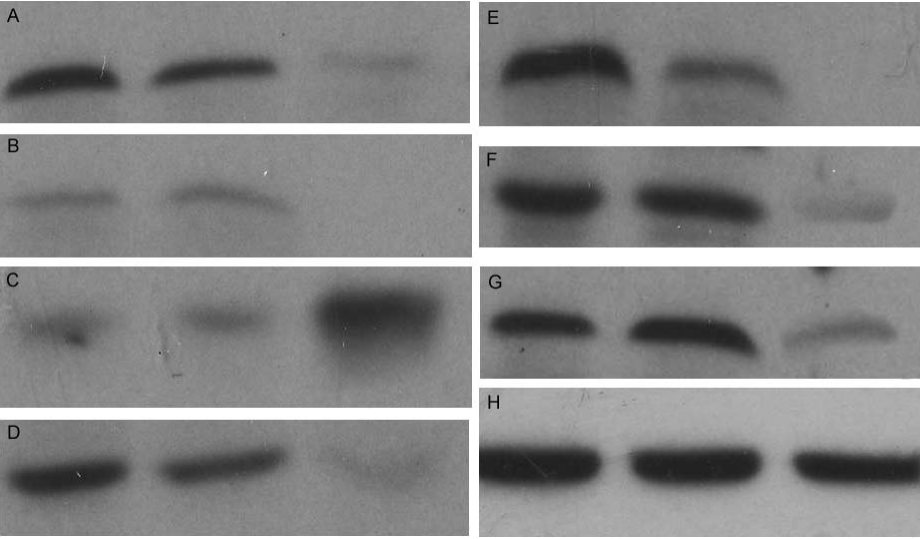
**Detection of MBD1 and differentially expressed proteins by Western blot analysis**. The lanes are as follows: left: BxPC-3/vector; middle: untreated BxPC-3; right: BxPC-3/MBD1-siRNA. A: MBD1: 70 kDa, B: stathmin: 19 kDa, C: tubulin beta2: 53 kDa, D: vimentin: 57 kDa, E: HSP A8: 70 kDa, F: GRP78: 78 kDa, G: hnRNP K: 65 kDa, H: β-actin: 42 kDa.

The siRNA sequences targeting MBD1 were successfully cloned into the pGCsi-U6/Neo/GFP plasmid. DNA sequencing was performed to confirm that the recombinant plasmid was constructed correctly (data not shown). The plasmid expressed green fluorescent protein (GFP) to allow observation of the transfected cells by fluorescent microscopy. Twenty-four hours after transfection, transfected BxPC-3 cells expressed GFP. Cells were selected with G418 (800 μg/mL) for approximately three weeks: positive cell clones were identified by fluorescent microscopy (Fig 1). Western blot analysis revealed that the expression of MBD1 in BxPC-3/MBD1-siRNA cells was significantly lower than in BxPC-3/vector and untreated BxPC-3 cells (Fig 2A). Quantitative RT-PCR confirmed that MBD1 in BxPC-3/MBD1-siRNA cells was downregulated about 14.76-fold than in control cells (Fig 5A).

### 2-DE analysis and differential expression of proteins

**Figure 3:**
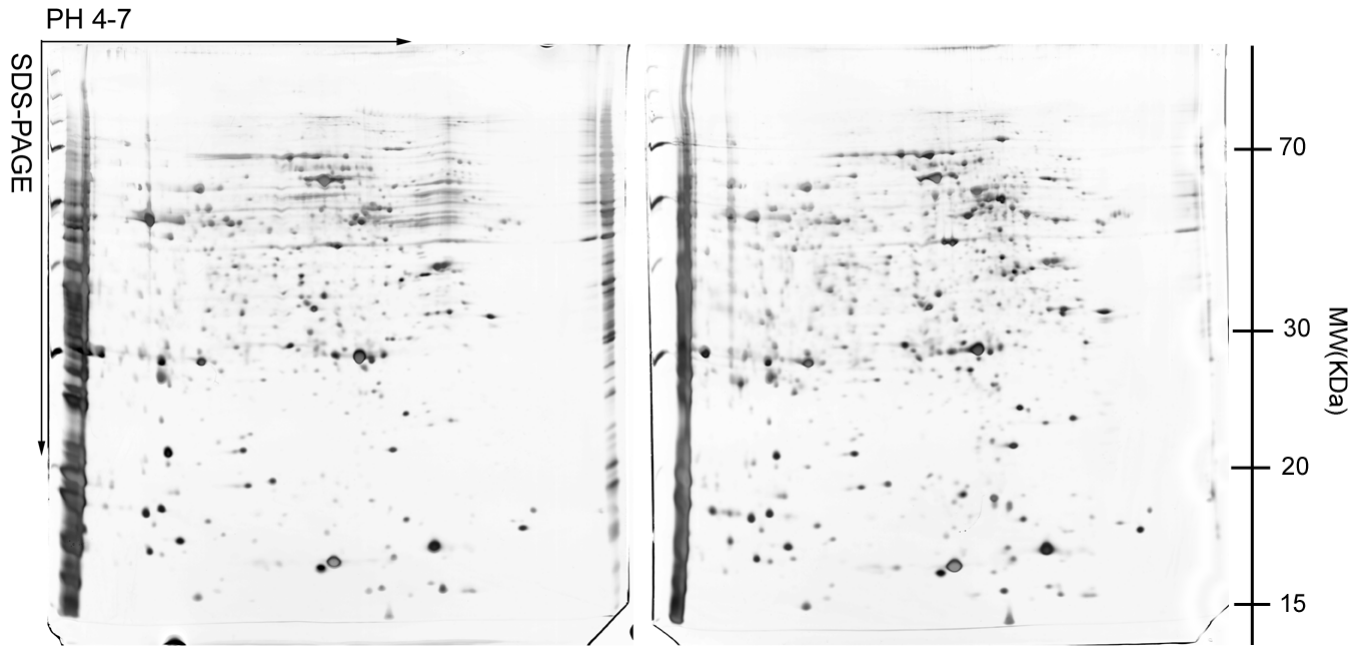
**2-DE maps from BxPC-3/MBD1-siRNA (left) and BxPC-3/vector (right) cell lines**. Figure shows two representative 2-DE maps in the pH range 4–7. 2-DE of each cell line was repeated at least three times.

**Figure 4:**
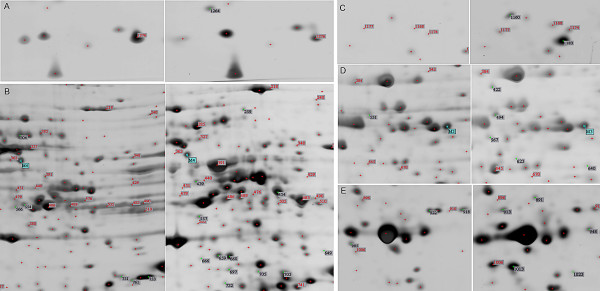
**Comparison of differentially expressed proteins between BxPC-3/MBD1-siRNA and BxPC-3/vector cell lines**. A, B, C, D, E: five areas of labeled protein spots on 2-DE gel. left panel: BxPC-3/MBD1-siRNA, right panel: BxPC-3/vector.

To investigate the changes in protein expression in pancreatic cancer after MBD1 knock-down, we performed a comparative proteomic study between BxPC-3/MBD1-siRNA and BxPC-3/vector cell lines. Fig 3 shows two representative 2-DE maps from BxPC-3/MBD1-siRNA and BxPC-3/vector cell lines in the pH range 4–7. Among the spots investigated, almost 30 proteins were differential expressed. But some of them were unknown proteins that could not be identified, and some of them with different PI and MW finally proved to be the same protein (such as 348 and 429, both identified as tubulin beta), which suggest different post-translational modification. Finally, 14 spots were identified as differentially expressed proteins between the BxPC-3/MBD1-siRNA and BxPC-3/vector cell lines. Using image analysis we found that five spots (429, 896, 384, 922, 363) were up-regulated and nine spots (391, 1177, 1264, 640, 557, 703, 649, 1023, 218) were down-regulated after the silencing of MBD1 using RNAi (Fig 4).

### Identification of differentially expressed proteins by MALDI-TOF-MS

The 14 differentially expressed protein spots were analyzed by MALDI-TOF-MS and identified by Mascot search. As shown in Table 2, the following five proteins were up-regulated: i) tubulin beta 2, ii) splicing factor arginine/serine-rich isoform 1, iii) ER-60 protease, iv) propyl 4-hydroxylase, beta subunit precursor (P4 hb) and v) EF hand domain family, member D2. The following nine proteins were down-regulated: i) 78 kDa glucose-regulated protein precursor (GRP 78), ii) Tollip (toll interacting protein), iii) heat shock 70 kDa protein 8 (HSP A8), iv) vimentin, v) stathmin 1, vi) cofilin 2, vii) heterogeneous nuclear ribonucleoprotein K (hnRNP K), viii) eukaryotic translation initiation factor 3 (eIF3), subunit 5, and ix) zinc finger protein ZFP-36.

**Table 2:** Proteins that showed differential expression after RNAi targeting MBD1

Spot No.	Accession No.	Protein Name	Protein MW	Protein pI	Protein Score	Fold change
429	**gi| 5174735**	tubulin, beta, 2	49808	4.79	68	↑4.05
896	**gi| 5902076**	splicing factor, arginine/serine-rich 1 isoform 1	27727.8	10.37	191	↑3.52
384	**gi| 1208427**	ER-60 protease	56760.8	5.98	140	↑4.09
363	**gi| 20070125**	prolyl 4-hydroxylase, beta subunit precursor	57058.6	4.76	70	↑4.06
922	**gi| 20149675**	EF hand domain family, member D2	26680.5	5.15	120	Only in BxPC-3/MBD1-siRNA
218	**gi| 14916999**	78 kDa glucose-regulated protein precursor (GRP 78) (Heat shock 70 kDa protein 5)	72377.5	5.07	249	↓4.02
1023	**gi| 6048243**	TOLLIP protein	23206.7	5.68	116	Only in BxPC-3/ vector
649	**gi| 48257068**	HSPA8 protein	64633.1	5.36	95	Only in BxPC-3/ vector
391	**gi| 62414289**	vimentin	53619.1	5.06	334	↓20.64
1177	**gi| 5031851**	stathmin 1	17291.9	5.76	90	↓3.48
1264	**gi| 14719392**	cofilin2	18724.8	7.66	74	Only in BxPC-3/ vector
640	**gi| 55958543**	heterogeneous nuclear ribonucleoprotein K	33954.7	5.54	74	Only in BxPC-3/ vector
557	**gi| 4503519**	eukaryotic translation initiation factor 3, subunit 5	37540.1	5.24	131	Only in BxPC-3/ vector
703	**gi| 141623**	Zinc finger protein ZFP-36	66639.8	8.99	65	Only in BxPC-3/ vector

### Validation of proteomics results

To verify the authenticity and reproducibility of the proteomics results, vimentin, stathmin, hnRNP K, GRP78, HSP A8 and tubulin beta2 were selected and confirmed by Western blot. As shown in Fig. 2, vimentin, stathmin, hnRNP K, GRP78 and HSP A8 were significantly down-regulated after MBD1 knock-down, while tubulin beta2 was up-regulated in BxPC-3/MBD1-siRNA, which were consistent with proteomics analyses.

**Figure 5:**
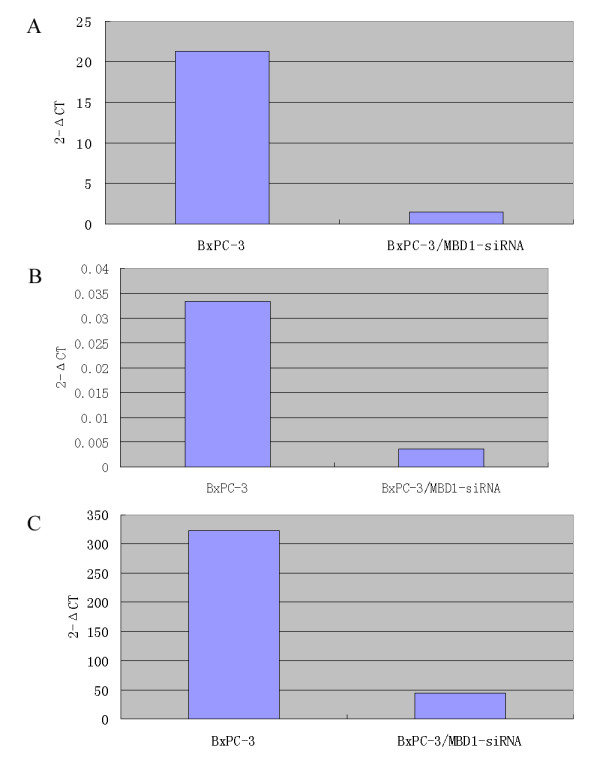
**Quantitative RT-PCR assay**. A: MBD1 in BxPC-3/MBD1-siRNA cells was downregulated about 14.76-fold after RNAi. B: In a short term RNAi experiment (48 hours after MBD1-siRNA vector transfection), vimentin was downregulated about 9.23-fold. C: In a short term RNAi experiment (48 hours after MBD1-siRNA vector transfection), GRP78 was downregulated about 7.14-fold.

In a short term RNAi experiment (two siRNA used), we found that 48 hours after MBD1-siRNA vector transfection, the mRNA level of vimentin and GRP78 were downregulated about 9.23-fold and 7.14-fold respectively by a quantitative RT-PCR assay (Fig 5B,C), which indicated that the changes in protein expression could be secondary to changes in cells caused by MBD1 knock-down.

## Discussion

MBD1 is a mammalian protein that binds symmetrically methylated CpG sequences and regulates gene expression in association with DNA methylation [2]. A powerful transcriptional repression domain (TRD) at the C terminus of MBD1 can actively repress transcription at a distance [4]. Thus, MBD1 may have an important role in the silencing of tumor suppressor genes that are hypermethylated at their promoter CpG islands in cancer cells [5,10].

Previously, we have shown that the gene expression of MBD1 is significantly increased in pancreatic carcinomas compared to normal pancreatic tissue, with a simultaneous decrease in the expression of some tumor suppressor genes (*CDH1, RB*) [11]. We also found that the protein and mRNA expressions of MBD1 in pancreatic carcinoma were significantly higher than in normal tissues. Furthermore, the expression of MBD1 correlated with lymph node metastasis [6]. However, the role of MBD1 during the development of pancreatic cancer is still unknown.

The present study combined a proteomic approach with RNAi technology to identify proteins associated with MBD1 function in pancreatic cancer. We successfully used RNAi technology to construct a recombinant MBD1-siRNA plasmid that stably knocked-down *MBD1 *gene expression in the BxPC-3 human pancreatic cancer cell line. We identified 14 differentially expressed protein spots between the two cell lines using MALDI-TOF-MS.

MBD1 affect gene expression according to the status of DNA methylation. Fujita N et al indicated that MBD1 acts as a transcriptional regulator depending on the density of methyl-CpG pairs through the cooperation of MBD, CXXC, and TRD sequences. MBD1v1 represses transcription preferentially from both unmethylated and sparsely methylated promoters, while MBD1v3 inhibits densely methylated but not unmethylated promoter activities. Moreover, they also found that MBD1 enhanced transcription from the some methylated promoter [2]. In our study, 9/14 proteins were downregulated and 5/14 proteins were upregulated after MBD1 knock-down, maybe related with different transcriptional regulation way of MBD1.

Among the 14 MBD1-associated proteins identified in this study, vimentin was found to be down-regulated 20-fold in the BxPC-3/MBD1-siRNA cell line compared to the control BxPC-3/vector cell. Vimentin, a major intermediate filament protein of mesenchymal cells, was found to be over-expressed in some human cancer tissues and associated with prognostic indicators [12,13]. Vimentin exon-1 sequences are methylated in most of colon cancer tissues and aberrant methylation of vimentin has become a novel molecular biomarker of colorectal cancer [14]. In the short term RNAi experiment, the mRNA level of vimentin was also down-regulated after MBD1 knock-down, indicated that MBD1 may involve in DNA methylation of vimentin and mediate the expression of vimentin in pancreatic cancer.

We also found that several members of the HSP family (GRP78 and HSP A8) were down-regulated in the absence of MBD1. GRP78 promotes tumor proliferation, survival, metastasis, and resistance to a wide variety of therapies [15,16]. Indeed, it has been shown that cancer cells adapt to chronic stress in the tumor microenvironment by inducing the expression of GRP78 [15,16]. Studies show that Heat shock proteins are methylated in mammalian cells and HSPs expressions are associated with aberrant methylation of promoter CpG islands [17-19]. In this study, the down-regulation of both GRP78 and HSP A8 after MBD1 knock-down, suggest that MBD1 may affect the expression of HSPs in pancreatic cancer through DNA methylation.

Many of the proteins that were found to be down-regulated by the knock-down of MBD1 have been shown to be over-expressed in certain cancers. For example, stathmin, a microtubule-destabilizing protein, is expressed at high levels in a wide variety of human cancers [20,21]. Inhibition of stathmin expression in malignant cells has been shown to interfere with cell cycle progression and abrogates the transformed phenotype [20,21]. hnRNP K, involved in regulating transcription, mRNA shuttling, RNA editing and translation, has been reported to be overexpressed in several human cancer tissues [22,23]. In colorectal cancer, patients who presented with hnRNP K-positive tumors had a poorer survival outcome [22,23]. EIF3 plays an essential role in the rate-limiting initiation phase of translation, and increased mRNA and protein levels have been detected in several human tumors. Furthermore, over-expression of eIF3 is reportedly a prognostic biomarker for poor clinical outcome [24]. Cofilin 2, an actin-depolymerizing factor that is mainly expressed in smooth muscle cells of collateral arteries, has been associated with angiogenesis [25]. Most of these proteins play an important role in tumorigenesis, some of them are prognostic biomarkers for human malignant tumors, and some were involved in methylation. For instance, different methylation sites in hnRNP K have been identified and shown to influence protein-protein interactions [26]. Stathmin also presents aberrant methylation in prostate cancer cell [27]. Down-regulation of these tumor-related proteins after MBD1-knock-down suggests that MBD1 may play a significant role in pancreatic cancer through regulating methylation.

Among the five proteins that were up-regulated, ER-60 protease and P4 hb are multifunctional proteins that belong to the protein disulfide isomerase family that is a chaperone protein and related with tumor cell migration and invasion [28-30]. Tubulin beta2, the subunit protein of microtubules, is present in the cell nuclei of a variety of cancers [31]. In colorectal cancer research, methylated DNA sequence within tubulin was identified [32]. Since these proteins were up-regulated after knock-down of MBD1, they might be novel MBD1-associated proteins in pancreatic cancer.

## Conclusion

We established a MBD1 knock-down BxPC-3 pancreatic cancer cell line using a stable RNAi method. The comparative proteomic approach is a useful strategy of proteomics to analyze and compare the differentially expressed proteins and identify novel target molecules in unknown pathways. These differential proteins may be associated with the function of MBD1, our findings provide some insight into the functional mechanism of MBD1 in the development of pancreatic cancer.

## List of abbreviations

MBD1: Methyl-CpG binding domain protein 1; RNAi: RNA interference; 2-DE: two-dimensional gel electrophoresis; MALDI-TOF: matrix-assisted laser desorption/ ionization time-of-flight; MS: mass spectrometry; SDS-PAGE: sodium dodecyl sulfate-polyacrylamide gel; NCBI: National Center for Biotechnology Information.

## Competing interests

The authors declare that they have no competing interests.

## Authors' contributions

CL, YC, CJ, JX, JL, DF and HJ made substantial contributions to conception, design, analysis, acquisition, interpretation of the data, as well as drafting and approving the final manuscript. QN, XY, and CB made substantial contributions to conception and interpretation of the data, critical revisions of the manuscript and final approval of the manuscript.

## Pre-publication history

The pre-publication history for this paper can be accessed here:


